# Perpetrator characteristics and firearm use in pediatric homicides: Supplementary Homicide Reports - United States, 1976 to 2020

**DOI:** 10.1186/s40621-024-00518-0

**Published:** 2024-08-12

**Authors:** Mark T. Berg, Ethan M. Rogers, Hannah Rochford

**Affiliations:** 1 Department of Sociology and Criminology, University of Iowa, 401 North Hall, Iowa City, IA 52242 USA; 2 Public Policy Center, University of Iowa, 605 E Jefferson Street, Iowa City, IA 52242 USA; 3 Department of Health Policy and Management, Texas A&M University, 212 Adriance Lab Rd, College Station, TX 77843 USA

**Keywords:** Pediatric homicide, Child homicide, Adolescent homicide, Perpetrator, Victimization, Homicide trends

## Abstract

**Background:**

Describe trends in perpetrator characteristics and firearm use in pediatric homicides across the United States.

**Methods:**

Multiply-imputed data from the Federal Bureau of Investigation’s 1976–2020 Supplementary Homicide Reports were used to estimate perpetrator characteristics (sex, age, and relationship to victim) and firearm use in pediatric homicides. Descriptive analyses were stratified by victim age group, sex, race, and five-year time periods.

**Results:**

Family members were the most common perpetrator of infant and toddler (ages 0–4) and child (ages 5–12) homicides, whereas acquaintances accounted for the majority of adolescent (ages 13–19) homicides. Perpetrator characteristics vary across victim sex and race, particularly among adolescents. Despite overall stability, there were changes in perpetrator characteristics from 1976 to 2020. There was a sustained increase in the proportion of homicides committed with a firearm. In 2016–2020, the proportion of firearm-involved homicides was an all-time high for infant and toddler (14.8%), child (53.1%), and adolescent victims (88.5%).

**Conclusions:**

Policy interventions that improve family stability and well-being may be most effective at preventing infant, toddler, and child homicides, whereas programs that target peer and community relationships, as well as policies that focus on firearm access, may be more crucial for preventing adolescent homicides.

## Background

Homicide is a leading cause of death among the pediatric population in the United States (US), with an estimated 38,362 children under age 18 killed in the US between 1999 and 2020 [1]. Children suffer higher homicide rates in the US than other developed nations [2, 3], and boys and Black children are disproportionately affected [1, 4]. Despite declines in the 1990s and 2000s, US pediatric homicide rates rose consistently since 2013 with a large spike during the COVID-19 pandemic [1, 4, 5]. Homicide victimization rates among individuals ages 10 to 19 increased by 39.1% from 2019 to 2020 [4]. Firearms are used in a large proportion of pediatric homicides, though not equally across pediatric age groups [1]. These facts underscore the importance of maintaining a robust empirical understanding of pediatric homicide. Surveillance is crucial to informing comprehensive evidence-based public health policy [4, 6]. In addition to victim characteristics, epidemiological studies of pediatric homicide should also provide national-level information about perpetrators. Understanding perpetrator characteristics (e.g., sex, age, and relationship to victim) is crucial to developing effective prevention strategies.

Recent research provides estimates of weapon type and perpetrator characteristics based on the National Violent Death Reporting System (NVDRS) [1]. NVDRS findings, however, may not be generalizable across the US due to a small number of states providing data, with as few as seven states participating in 2003 and less than twenty states participating through 2014 [7]. For example, the 9,881 homicide victims aged 0 to 17 years examined in one NVDRS analysis represent approximately one third of all US victims in that age group during the study period (2003–2019) [1]. There may be systematic differences in pediatric homicide characteristics between states that did and did not participate in the NVDRS. Furthermore, the NVDRS is limited in its ability to examine *trends* in homicide circumstances. Because participating states were added incrementally since 2003, it is difficult to assess whether temporal variation in homicide circumstances results from actual changes in homicides or the addition of states at different times [6, 8]. Moreover, the NVDRS data do not encompass the youth violence surge of the late 1980s and early 1990s. Estimates from national-level data systems, whose designs are consistent across decades, can be harnessed to document temporal changes in the characteristics of pediatric homicide perpetrators [9].

Previous studies indicate that homicide risk varies across pediatric age group, with risk peaking in early childhood and late adolescence [1, 10]. These patterns might reflect developmental differences in social engagement and independence. Toddlers are highly dependent on caregivers and not able to avoid conflict-ridden home environments or communicate their own maltreatment [11], whereas adolescents are increasingly independent and forge ties to similar age peers outside of the home, leaving them at greater risk of victimization from non-familial others [12]. The extent to which patterns of developmentally-dependent risk have been consistent over time remains unknown. Similarly, there is limited knowledge of how patterns of pediatric homicide perpetration vary across victim sex and race [13, 14]. It is also not clear how the perpetrator use of firearms differs by victim characteristics, and whether these patterns have varied over time.

As such, the current study’s primary objective was to describe perpetrator characteristics and firearm use in pediatric homicides across the US over four and a half decades using a nationally representative data system. A secondary objective was to describe trends in perpetrator characteristics and firearm presence by the age, sex, and race of pediatric victims. This descriptive information will add to the evidence on the changing epidemiology of pediatric homicide in the US [15–17]. Documenting national patterns in a longitudinal context can indicate how pediatric homicide perpetration intersects with demographic characteristics of victims, and how perpetrator characteristics change over time. Through this understanding, critical opportunities for public health intervention can be identified and implemented in an equitable manner.

## Methods

### Data source

We used nationally representative data from the Federal Bureau of Investigation’s (FBI) Supplementary Homicide Reports (SHR) to examine the perpetrator characteristics and firearm presence in pediatric homicide victimizations during 1976 to 2020. As part of the FBI’s Uniform Crime Reporting (UCR) program, the SHR provides information on criminal homicides known to US law enforcement agencies and submitted to the FBI program. A strength of the SHR is that it provides information on perpetrator characteristics. Neither the National Vital Statistics System (NVSS) Multiple Mortality File nor the restricted National Death Index provide details regarding suspected perpetrators or the circumstances surrounding the death. As such, researchers maintain that the SHR data are “better suited for understanding the circumstances surrounding homicide incidents, including. characteristics of the offender, and the relationship between the victim and the offender.” [18, p.4] Additionally, the SHR data consistently shows similar homicide rate trends at the national level when compared to the NVSS data [8, 18]. The NVDRS offers information on homicide incidents but its design limits its utility for generating nationally representative long-term homicide estimates [9].

While the SHR provides a comprehensive account of the nation’s pediatric homicide burden, the data system is not without limitations. Not every US law enforcement agency reports its data to the SHR each year, resulting in what is known as “unit missingness” in the series. When benchmarked to the FBI’s UCR homicide victimization counts, 7% of homicide incidents are not recorded in the 1976–2020 SHR data. Also, reported homicide incidents contain missing case information or “item missingness.” [19] Based on 1976–2005 SHR data, over one quarter of homicide incidents were missing information on the age, sex, and race of the perpetrator [19].

To adjust for cases not recorded in the SHR and to supplement incomplete homicide case data, we use a multiply-imputed 1976–2020 SHR series [20], developed from a validated two-stage multivariate missing data strategy [19]. To account for unit missingness, SHR homicide victimization counts were weighted to match the FBI’s UCR counts by year and state. The age, sex, and race distributions of victims were also adjusted to conform to these demographic distributions provided in the National Center for Health Statistics mortality data. To account for item missingness in the SHR, a Bayesian Imputation Posterior method was used, under the assumption that the missing data are missing at random. A log-linear model generated five sets of imputations for missing victim, perpetrator, and incident characteristics. The multiply-imputed SHR database includes a weight to apply when generating estimates from the five imputed data sets. Estimates based on multiply-imputed SHR data are important for research because they are “less prone to biased estimates and deflated standard errors” than the non-imputed SHR data [19, p.76]. The multiply-imputed SHR database has been widely used in descriptive research on population-level homicide trends [21, 22] and in empirical assessment of firearm ownership [23, 24] and state firearm laws on homicide [25].

### Analysis

Perpetrator characteristics included sex (male, female), age (under 18, 18 plus), and relationship to victim, coded as family (e.g., parent, sibling), acquaintance (e.g., neighbor, friend), stranger, and intimate partner (e.g., spouse, ex-spouse, boyfriend, girlfriend). In incidents with multiple perpetrators, the recorded characteristics reflect the first perpetrator in the file. Note that the SHR data structure limits the accuracy of estimates regarding characteristics of multiple victim incidents. Specifically, the perpetrator relationship is fixed for all victims in a single incident: so for each victim, the perpetrator relationship refers only to the first victim. Thus, the recorded perpetrator-victim relationship for additional victims (4.7% of all victims) may be inaccurate. We retain these incidents for the present analysis but note the limitations for interpretive purposes. For example, the perpetrator of a toddler homicide may be classified as an intimate partner if both a toddler and parent were killed by the parent’s significant other and the parent was the first victim in the file. As a result, there may be incidents in which a perpetrator of a toddler homicide may be coded as an “intimate partner.” In these rare cases, “intimate partner” would be more appropriately interpreted as “intimate partner of one of the victims.”

We generated sixteen distinct perpetrator profiles = perpetrator sex (male, female) x perpetrator age (under 18, 18 plus) x perpetrator relationship (family, acquaintance, stranger, intimate partner). We estimated the percent of homicide victimization incidents involving each profile. We recoded the indicator of weapon type as firearm or non-firearm (e.g., knife, blunt object, personal weapon).

The descriptive analyses were stratified by victim age group (infants and toddlers under 5 years, children 5 to 12 years, and adolescents 13 to 19 years), victim sex (male, female), and victim race (Black, White). Age groups were based on evidence that pediatric homicide victimization rates tend to be highest in early childhood and late adolescence, and lowest among school-aged children [10]. To assess change, the descriptive analyses were portioned by five-year periods. Annual homicide estimates were aggregated into nine five-year periods (e.g., 1976–1980) due to the small cell counts in single-year estimates. Reliable information on homicide counts by victim ethnicity are not provided in the SHR database and are not imputed in the data [19].

## Results

### Infant and toddler victims, age 0–4

**Fig. 1:**
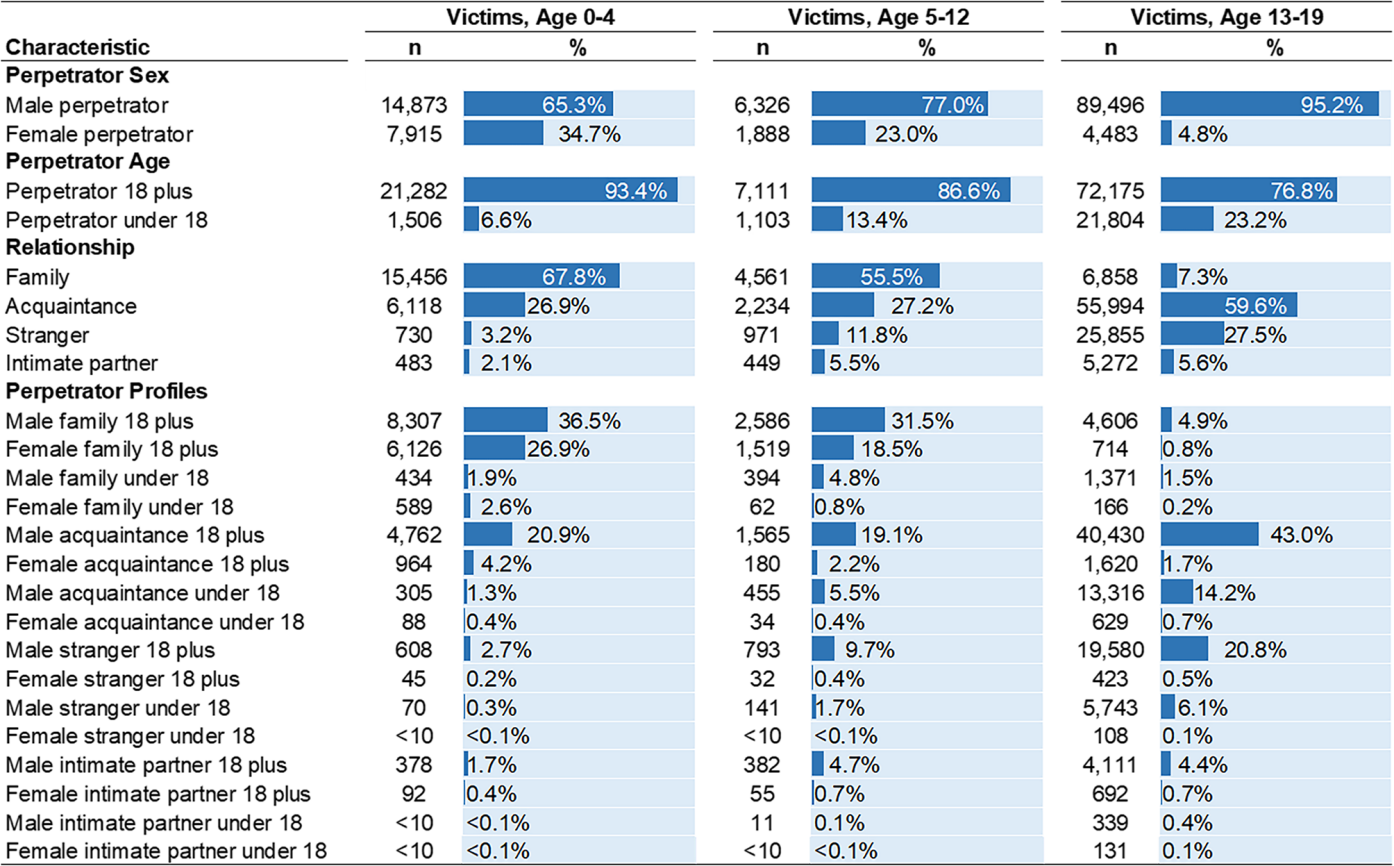
Perpetrator Characteristics in Pediatric Homicides by Victim Age Group. Number (*n*) and percentages (%) are presented. Estimates calculated from Supplementary Homicide Reports, United States, 1976 to 2020

**Perpetrator characteristics**. Most infant and toddler homicides are perpetrated by males (65.3%), individuals 18 years and older (93.4%), and family members (67.8%, Fig. 1). Approximately 34.7% are perpetrated by females, and 26.9% by acquaintances. Three perpetrator profiles account for over 80% of all infant and toddler homicide victimizations: male family members 18 years and older (36.5%), female family members 18 years and older (26.9%), and male acquaintances 18 years and older (20.9%).

**Fig. 2:**
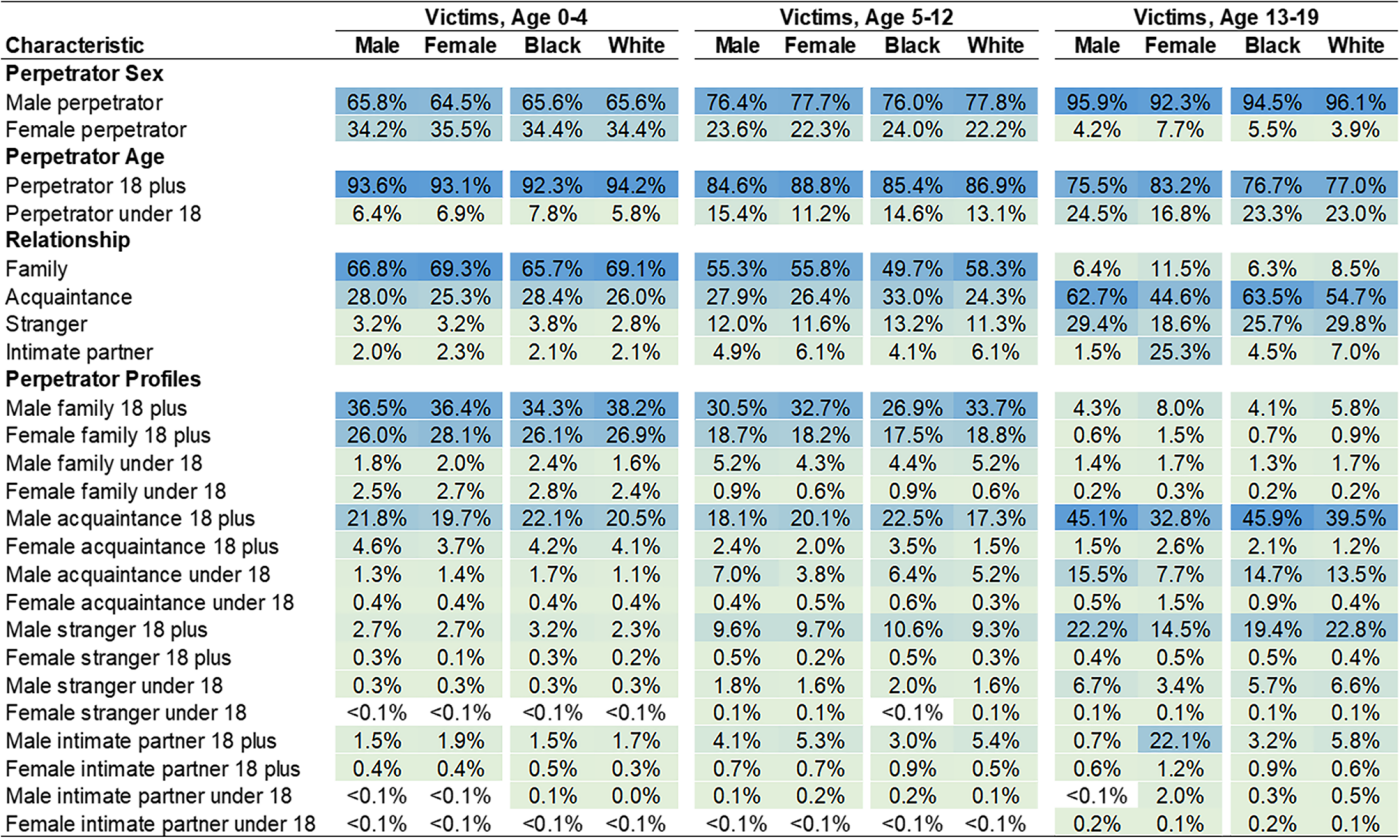
Perpetrator Characteristics in Pediatric Homicides by Victim Age Group, Victim Sex, and Victim Race. Percentages (%) are presented. Estimates calculated from Supplementary Homicide Reports, United States, 1976 to 2020

**Perpetrator characteristics by victim sex and race**. Among infant and toddler homicide victimizations, perpetrator characteristics are similar across victim sex and victim race (Fig. 2). For both male and female infant and toddler victims, the most common perpetrators were males (65.8%, 64.5%), individuals 18 years and older (93.6%, 93.1%), and family members (66.8%, 69.3%). For both Black and White infant and toddler victims, the most common perpetrators were males (65.6%, 65.6%), individuals 18 years and older (92.3%, 94.2%), and family members (65.7%, 69.1%). Regardless of victim sex or race, male family members 18 years and older were most often responsible for infant and toddler homicides, followed by female family members 18 years and older, and male acquaintances 18 years and older.

**Fig. 3:**
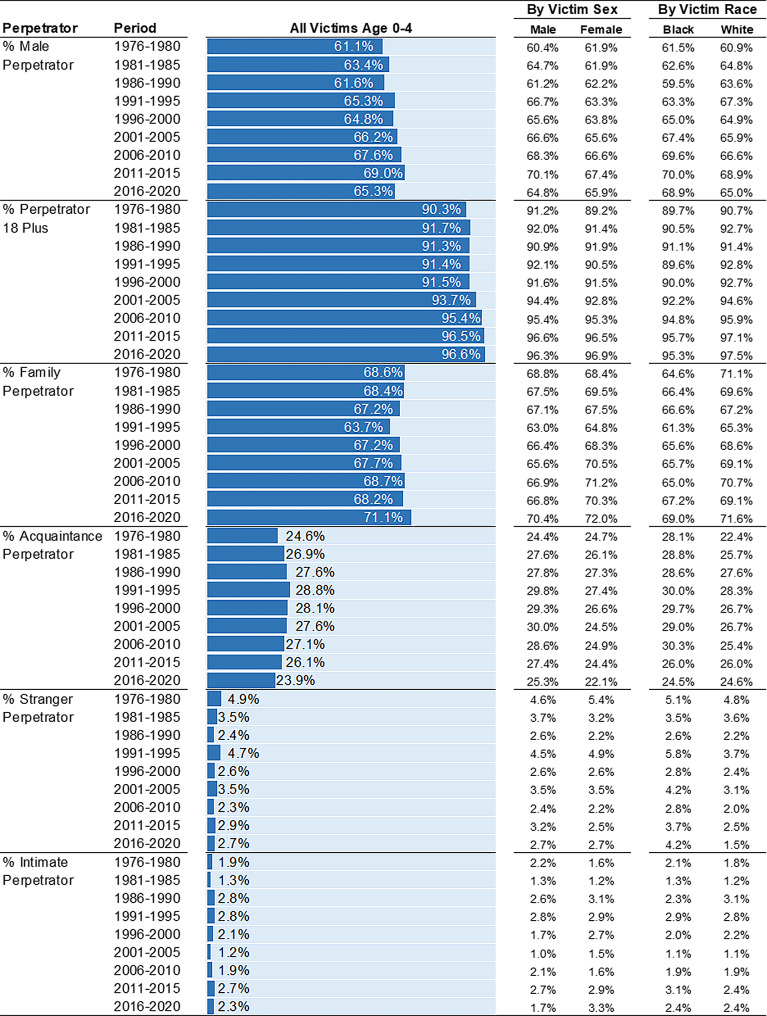
Perpetrator Characteristics among Homicide Victims Age 0–4 by Victim Sex, Victim Race, and 5-Year Period. Percentages (%) are presented. Estimates calculated from Supplementary Homicide Reports, United States, 1976 to 2020

**Perpetrator characteristics over time**. The percentage of infant and toddler homicides perpetrated by males increased over time (Fig. 3). Compared to 61.1% in 1976–1980, two-thirds (65.3%) of infant and toddler homicides were perpetrated by males in 2016–2020. There was a slight increase in the proportion of killings attributed to perpetrators 18 years and older. Approximately 96.6% of infant and toddler killings were perpetrated by individuals 18 years and older in 2016–2020, up from 90.3% in 1976–1980. Relationship patterns remained largely stable across time, with 67.2–71.1% of infant and toddler homicides perpetrated by family members in all but one five-year period. In 1991–1995, 63.7% of homicides were perpetrated by family members. Across every five-year period, the proportion of infant and toddler homicides perpetrated by a friend or acquaintance ranged between 23.9% and 28.8% and the proportion perpetrated by strangers ranged between 2.3% and 4.9%.

**Fig. 4:**
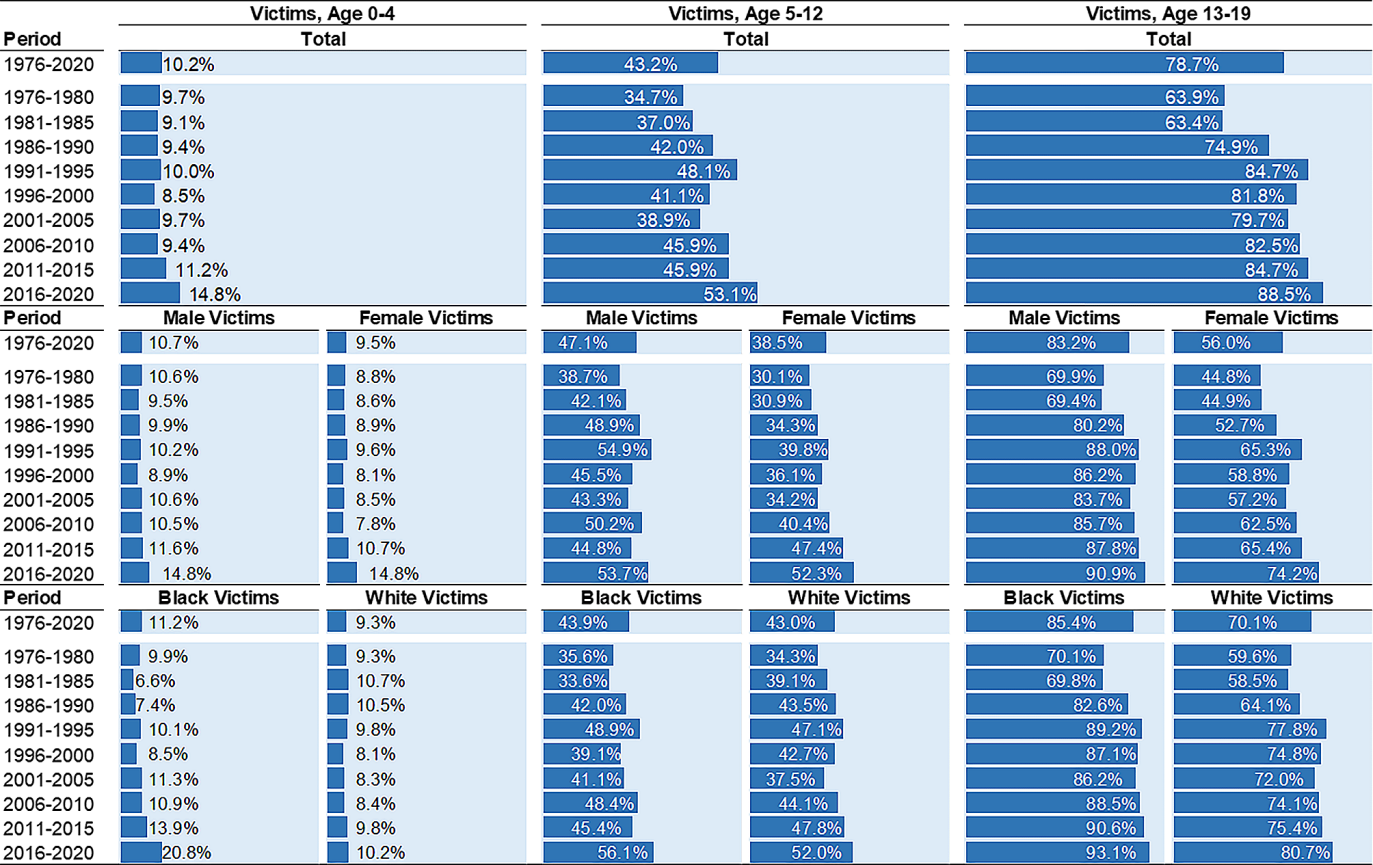
Firearm Presence in Homicide Victimizations, by Victim Age Group, Victim Sex, Victim Race, and 5-Year Period. Percentages (%) are presented. Estimates calculated from Supplementary Homicide Reports, United States, 1976 to 2020

**Firearm presence**. In 1976–2020, one-in-ten infant and toddler homicides were perpetrated using firearms (Fig. 4). Firearm use was similar in both male (10.7%) and female (9.5%) victimizations, as well as both Black (11.2%) and White (9.3%) victimizations. This summary, however, masks key trends. The share of firearms in infant and toddler homicides was stable from 1976 to 1980 to 2006–2010, but then steadily increased from 9.4% in 2006–2010 to 14.8% in 2016–2020 for both male (10.5–14.8%) and female (7.8–14.8%) homicide victimizations. Among White infant and toddler victims, firearm use has not changed. The proportion of firearm involved Black infant and toddler victimizations nearly doubled from 2006 to 2010 (10.9%) to 2016–2020 (20.8%). In 2016–2020, the proportion of Black infant and toddler victimizations (20.8%) was more than twice that of their White counterparts (10.2%).

### Child victims, age 5–12

**Perpetrator characteristics**. Males (77.0%) and individuals 18 years and older (86.6%) overwhelmingly perpetrated child homicides (Fig. 1). Over half were perpetrated by family members (55.5%), and 27.2% by acquaintances. Similar to infant and toddler homicides, the most common perpetrator profiles in child homicides were male family members 18 years and older (31.5%), male acquaintances 18 years and older (19.1%), and female family members 18 years and older (18.5%). Roughly 9.7% of children were killed by male strangers 18 years and older.

**Perpetrator characteristics by victim sex and race**. The descriptive statistics for perpetrator and relationship profiles are similar for male and female child homicide victims. For example, males perpetrated 76.4% of male and 77.7% of female child homicide victimizations. The patterns of perpetrator sex and perpetrator age are similar for Black and White victims (Fig. 2). Patterns of perpetrator-victim relationships differ by racial groups. For example, a larger fraction (33.0%) of Black child victims were killed by acquaintances than their White counterparts (24.3%).

**Fig. 5:**
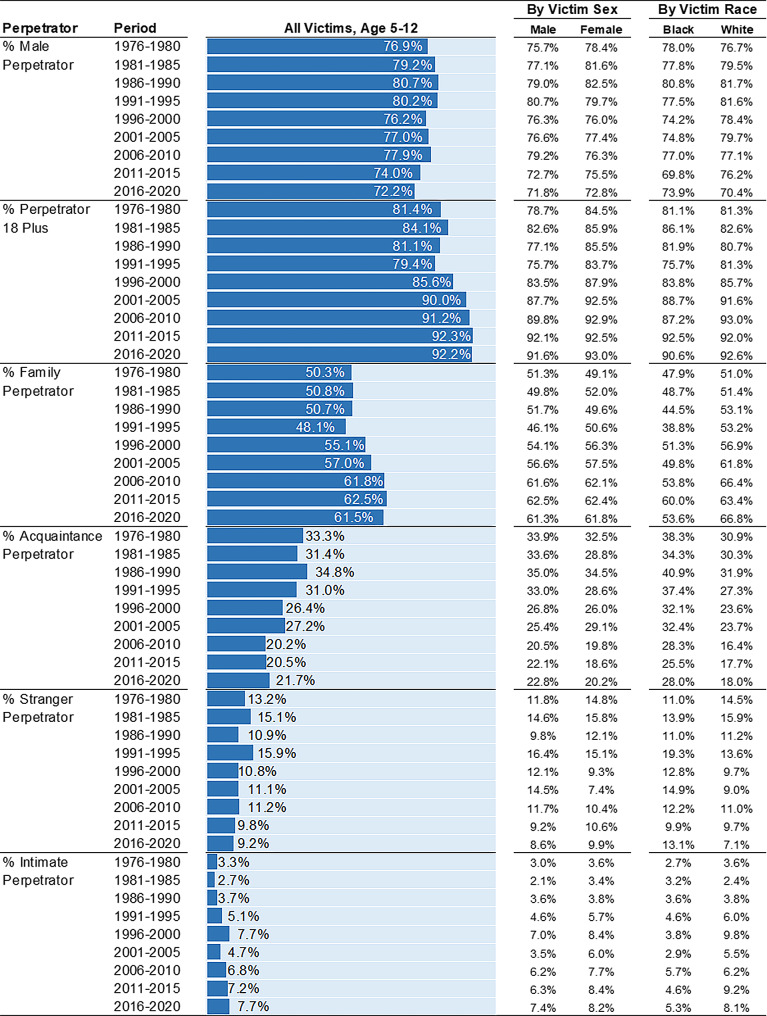
Perpetrator Characteristics among Homicide Victims Age 5–12 by Victim Sex, Victim Race, and 5-Year Period. Percentages (%) are presented. Estimates calculated from Supplementary Homicide Reports, United States, 1976 to 2020

**Perpetrator characteristics over time**. Among child homicides, the proportion of male perpetrators, perpetrators under 18 years, and acquaintance perpetrators has decreased over time (Fig. 5). For instance, in 2016–2020, 72.2% of child homicides were perpetrated by males – the smallest percentage across all periods. In the late 1980s and early 90s, approximately 80% of child homicides were perpetrated by males. From 1976 to 1980 to 2016–2020, the proportion of child homicides perpetrated by acquaintances decreased from 33.3 to 21.7%. But, the proportion perpetrated by family members increased (50.3–61.5%). Individuals 18 years and older perpetrated 92.2% of child homicides in 2016–2020, up from 81.4% in 1976–1980.

**Firearm presence**. Approximately 43% of child homicides in 1976–2020 involved a firearm (Fig. 4). Firearms are more often used in male (47.1%) than female child victimizations (38.5%). Similar proportions of both Black (43.9%) and White children (43.0%) are killed by firearms. Firearm use in child homicides increased from 1976 to 1980 (34.7%) to 2016–2020 (53.1%). The increasing prevalence of firearm homicides from 1976 to 1980 to 2016–2020 occurred for both male (38.7–53.7%) and female victims (30.1–52.3%), as well as Black (35.6–56.1%) and White victims (34.3 to 52.0%).

### Adolescent victims, age 13–19

**Perpetrator characteristics**. Almost all adolescent homicides in 1976–2020 were perpetrated by males (95.2%), compared to 4.8% perpetrated by females (Fig. 1). Over three-quarters were perpetrated by individuals 18 years and older (76.8%), 59.6% by acquaintances, and 27.5% by strangers. Just 7.3% of adolescent homicides were perpetrated by family members, compared to infant and toddler homicides where family members account for the majority of killings. When examining perpetrator profiles of adolescent homicides, male acquaintances 18 years and older are the most common perpetrators (43.0%), followed by male strangers 18 years and older (20.8%), and male acquaintances under 18 years (14.2%).

**Perpetrator characteristics by victim sex and race**. The proportion of adolescent homicides perpetrated by males is similar across victim sex (Fig. 2) – males perpetrate 95.9% and 92.3% of killings of male and female adolescents, respectively. Perpetrator age, however, differs for male and female adolescent victims. Individuals under 18 years account for roughly 16.8% of female adolescent homicide victimizations and roughly 24.5% of male homicides. There are also differences in relationship characteristics across victim sex. Compared to female adolescent victims, male adolescent victims are more often killed by acquaintances (62.7% vs. 44.6%) and strangers (29.4% vs. 18.6%). Just 1.5% of male adolescent victims were killed by intimate partners compared to one quarter (25.3%) of female adolescent victims. Across victim race (Fig. 2), there is little variation in the perpetrator characteristics of adolescent homicides. Compared to White adolescents, Black adolescents are just as often killed by males (94.5% vs. 96.1%) and individuals 18 years and older (76.7% vs. 77.%), indicating similarities in risk.

**Fig. 6:**
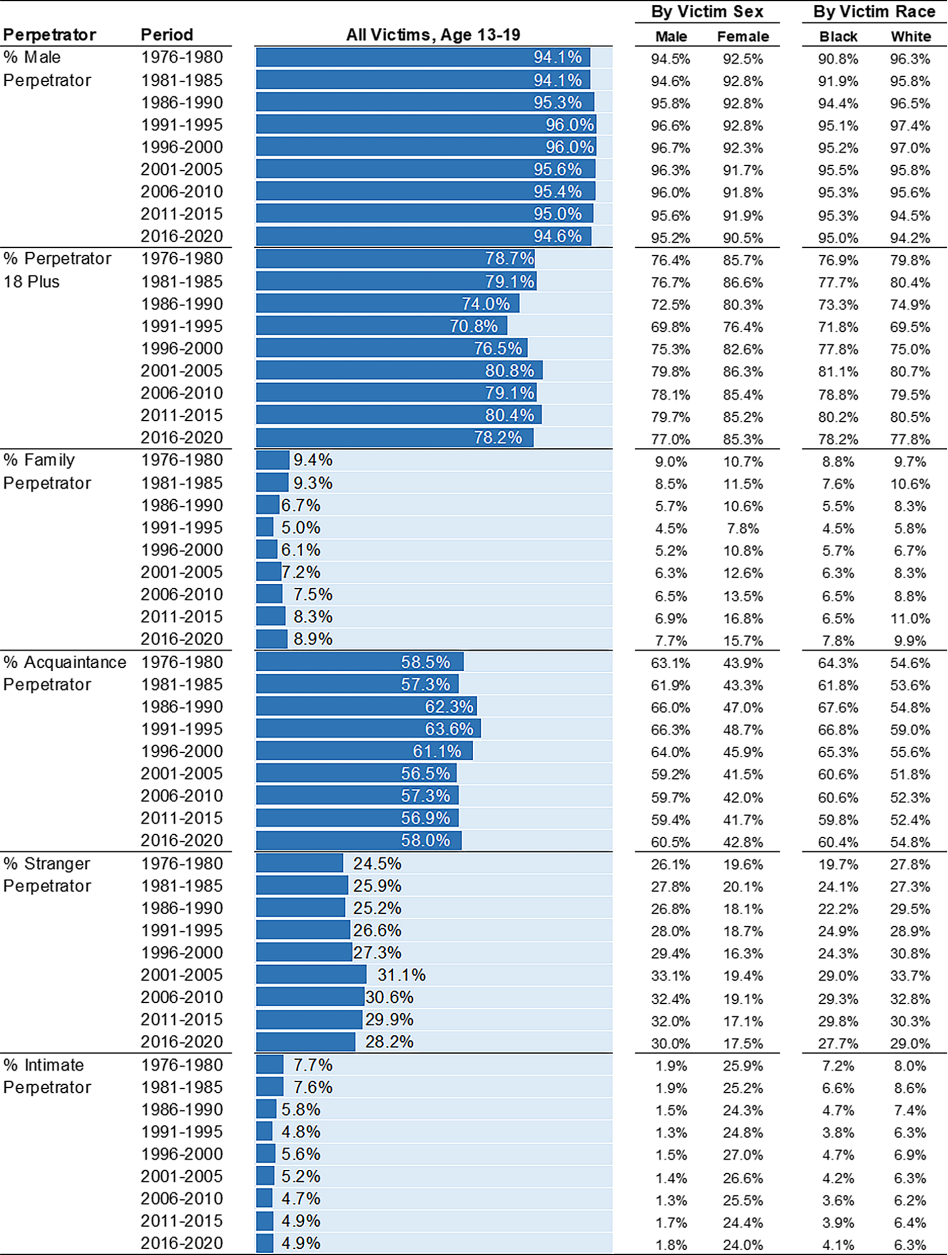
Perpetrator Characteristics among Homicide Victims Age 13–19 by Victim Sex, Victim Race, and 5-Year Period. Percentages (%) are presented. Estimates calculated from Supplementary Homicide Reports, United States, 1976 to 2020

**Perpetrator characteristics over time**. Overall, the perpetrator characteristics of adolescent homicides show marked stability over time (Fig. 6). Comparing 2016–2020 to 1976–1980, a similar proportion of adolescent victims are killed by male perpetrators (94.6% vs. 94.1%), individuals 18 years and older (78.2% vs. 78.7%), and acquaintances (58.0% vs. 58.5%). These trends are observed for both male and female, and Black and White adolescents. A closer look, however, reveals that the share of adolescent homicides perpetrated by individuals under 18 years was higher in the late 1980s and early 1990s than all other five-year periods.

**Firearm presence**. During the study period, more than three-quarters (78.7%) of adolescent victims were killed by firearms (Fig. 4). The proportion perpetrated by firearms is higher among male (83.2%) than female victims (56.0%), and higher among Black (85.4%) than White victims (70.1%). Over time, the firearm share has climbed from 63.9% in 1976–1980 to 88.5% in 2016–2020. Increases in firearm use were particularly pronounced among female victims – an increase from 44.8% in 1976–1980 to 74.2% in 2016–2020. By 2016–2020, more than 90% of male adolescent victims (90.9%) and Black adolescent victims (93.1%) were killed by firearms.

## Discussion

The US pediatric homicide burden continues to be a focus of public health science and practice. The findings of this study provide a national-level overview of trends in perpetrator characteristics and firearm involvement in infant and toddler, child, and adolescent homicides. The findings add to the body of evidence on the changing epidemiology of pediatric homicide perpetration in the US [1, 16, 26].

Results reveal developmental differences in pediatric homicide circumstances, notably the perpetrators’ sex, age, relationship to victim, and firearm use. Perpetrator characteristics appear to differ across pediatric groups in ways that align with developmental changes in family dependency and interaction, peer and romantic relations, and age-related role independence [27]. For instance, the most common perpetrators of infant and toddler killings and child killings were male and female family members 18 years and older. Males were primarily responsible for pediatric homicides regardless of victim age. By contrast, female family members 18 years and older were responsible for more than a quarter of all infant and toddler killings, compared to less than 1% of adolescent killings.

Future research should continue to examine the contextual and situational circumstances that precipitate infant and child killings, including the motives for these killings. Some empirical evidence suggests that some infant and child homicides result from extreme and harsh discipline at the hands of caregivers, including unrelated adults [28–31]. Other research focused on homicides perpetrated across weapon types suggests infant and toddler killings are commonly perpetrated by a parent or mother’s male companion, and are often precipitated by caregiver abuse and neglect [1, 32]. Case studies on homicide-suicide incidents involving infant and child victims have shown that an argument or conflict between adult intimate partners, often in the context of separation, divorce, and custody disputes, is a predominant triggering characteristic of infant and child killings by their caregivers [33]. It is also important for research to examine the role of caregiver mental health in infant and child homicides, as evidence from the United Kingdom suggests that perpetrators with psychiatric disorders are over-represented in child homicide case samples [34].

The findings from the current study are consistent with past research based on smaller samples, which demonstrate age-related patterns in pediatric killings [35]. Patterns of adolescent killings reflect the developmental shift away from dependency on the family and the growing role of peer networks [27, 36]. Their growing exposure to strangers and peers elevates their risk of suffering lethal violence by members of these groups while decreasing their level of risk from family [37]. Indeed, acquaintances, such as friends and peers, accounted for the largest fraction of adolescent killings. Perpetrators under age 18, particularly male friends and acquaintances, were responsible for a larger fraction of adolescent homicide victimizations relative to other groups. The male-dominated nature of adolescent homicides is noteworthy: in any five-year period, more than nine-in-ten adolescent homicides of male, female, Black, and White victims were perpetrated by males. To understand age-related perpetration risk, research should examine the causes of pediatric homicide victimization, with a focus on developmental context, family processes, peer relationships, school characteristics, and neighborhood environments [38, 39]. For instance, several studies on non-lethal violence have shown that many interpersonal disputes among similarly aged peers, particularly young men, often erupt from conflicts over social status (e.g., respect), including concerns about projecting masculinity [40, 41]. These incidents spillover from neighborhoods into schools and vice-versa and escalate into increasingly serious incidents that spread much like a contagion process through adolescent social networks [42, 43]. These events are facilitated by ambient stressors of social environmental adversity, legal cynicism, and economic marginalization [44].

The findings also highlight group disparities in pediatric homicides, which should inform efforts to maximize the impact of violence prevention programming. Family members 18 years and older were the most responsible for infant and toddler killings. A similar pattern was observed for child homicides. Black children and adolescents, however, had a higher risk of lethal victimization from acquaintances relative to their White counterparts. Given these facts, etiological research should attempt to clarify this racial disparity in the perpetration of child killings. As researchers observe, Black communities are disproportionately affected by the concentration of acute social and environmental stressors that are particularly taxing for families including caretakers of young children. These stressors theoretically combine to create the motivating conditions for violence [45, 46]. For instance, related research finds that acute social stressors in the family (e.g., economic insecurity) including limited access to healthcare resources are risk factors for non-fatal child maltreatment [47]. The disparity in child killings perhaps reflects the enduring racial disparity in exposure to adverse conditions.

The findings also show that female adolescents were more likely than males to be killed by intimate partners, a disparity that also warrants ongoing research to inform prevention [48]. For instance, research is needed to understand why the adolescent gender gap in intimate partner homicide does not generally align with the gap in rates of non-fatal intimate partner violence [49]. Specifically, research shows that intimate partner homicide victimizations rates are significantly higher among girls than boys [32, 50, 51]. Furthermore, studies in the US and abroad find that the gender gap in rates of non-fatal less serious intimate partner victimization are narrower than homicide rates [52, 53]. In fact, some studies show that adolescent and young adult boys experience higher rates of intimate partner victimization than girls [54, 55]. Eisner recently noted in a review of the literature that research increasingly finds that girls perpetrate partner violence more or equally frequently than boys [56]. As the level of injury or seriousness increases, the gender gap becomes more pronounced with girls experiencing higher rates of injurious partner violence. Why boys kill female partners at higher rates is not clear, though research shows that male and female perpetrated non-fatal partner violence have overlapping but unique risk factors [57]. This research should explore if the two forms of violence originate from different motives and reveal something about the normative conditions responsible for fatal intimate partner violence [50]. Developing a greater understanding of the causes of the puzzling gender differences in rates of non-fatal and fatal intimate partner violence would thus reveal important clues about the motivations behind the uneven burden of fatal partner violence felt among girl victims [58].

Although the circumstances of pediatric homicides remained rather stable from 1976 to 2020, there were changes that causal research should attempt to explain. Moderately larger proportions of infant and toddler killings and child killings are committed by individuals 18 years and older than in years prior. Females and family members account for a larger fraction of child homicides in recent years than in the 1980s and 1990s. Researchers should isolate the factors that explain the temporal rise in the proportion of child homicides perpetrated by individuals 18 years and older and family members. Perhaps, as criminologists have suggested, these trends partly correspond with emerging family stressors including economic hardship in combination with changes in family structure since the late 1970s [59, 60]. Around the early 1990s, we observed a significant but transitory drop in the proportion of adolescent homicides perpetrated by individuals 18 years and older. This decline corresponds to the well-documented national surge in adolescent homicide victimizations during the early 1990s perpetrated by adolescents, particularly against Black victims [61, 62]. A large body of research has shown that this surge in youth killings among Black adolescents occurred in the context of a national economic downturn paired with high inflation that limited economic prospects in the formal labor market and amplified adverse conditions already established by racialized poverty and economic marginalization [45, 59, 60, 63]. The literature also suggests small portable firearms proliferated during this era due in part to their growing availability and affordability and their value in illicit markets [64]. Numerous studies of crime trends across large US cities have found that, as economic conditions improved in the late 1990s and early 2000s, homicide rates among Black adolescents dropped steadily - though racial disparities in youth homicide persist owing to a legacy of disenfranchisement and racialized poverty [59, 65].

The growing role of firearms in lethal violence figures prominently in discussions about the prevention of pediatric homicides. The current findings illuminate developmentally patterned variation in firearm killings as well as group disparities. For every victim age group, there was a sustained rise in the proportion of homicides committed with a firearm through the decades under investigation. Infant and toddler homicides are the least likely to be committed with firearms relative to other pediatric groups. This fraction, however, has climbed to an all-time high in the recent 5-year period (14.8%) with Black infants and toddlers experiencing *twice the burden* as White infants and toddlers. Firearm use was also higher in Black than White adolescent homicide victimizations across all five-year periods. The racial disparities in firearm killings highlights the persistent and rising health inequalities of firearm violence which disproportionately affect Black individuals [66]. In addition to increased risk of firearm homicide victimization, male Black children and adolescents are more likely to be victims of nonfatal firearm assault and to be exposed indirectly to community firearm violence, including witnessing or hearing a shooting [14]. More broadly, these patterns of firearm killings underscore the need for research on the conditions responsible for the increased involvement of firearms in pediatric homicides, including ownership patterns and permissive firearm laws around accessibility. Recent evidence suggests safe firearm storage practices are effective at reducing firearm related injury in pediatric populations [67]. There is also evidence suggesting the capabilities of firearms have become increasingly lethal (e.g., magazine size, portability, firing capacity) which may have contributed to increases in the rate of firearm killings, independent of the availability of firearms [68, 69].

### Policy implications

Altogether these findings highlight the importance of implementing prevention models that are developmentally sensitive, which target the life-course conditions of infants and toddlers, children, and adolescents that involve high violence risk. This would involve utilizing prevention models tailored to the persons or relationships — whether family members, intimates, or peers — which account for the greatest risk of violent death. Accordingly, policy interventions focused on supporting family stability and well-being (e.g., paid family leave [70], affordable childcare [71, 72]) may be particularly influential for preventing infant, toddler, and child homicides, including programs that target corporal punishment and abuse [73] and that promote positive parenting practices [74, 75]. Also, programs that position daycare and school staff to respond to maltreatment may be effective at reducing toddler and child homicides [11].

Such family-centered interventions, however, may not sufficiently address the risk circumstances of all pediatric homicide victim groups. For example, because the proportion of child homicides perpetrated by friends and acquaintances was higher for Black children across all five-year periods, family-centered policies may be disproportionately protective for White children. These programs may also not provide significant benefit for adolescent homicides as these are largely perpetrated by friends, acquaintances, and strangers. Programs to reduce homicides during adolescence should perhaps instead target peer and community relationships. For example, there may be value in implementing policies which promote safe school environments [76], foster positive peer relations, teach peaceful conflict resolution strategies [77], decrease high risk behaviors (e.g., gang membership, weapon carrying) [78], and focus on improving local conditions to increase community trust. Programs that focus on the prevention of intimate partner violence among young people may be effective at reducing partner homicides among females [79]. Evidence-based firearm access policies (e.g., safe storage laws, expanded background checks, permit-to-purchase requirements, and minimum age laws) [80], may also be particularly effective homicide prevention strategies for older pediatric groups given the substantially larger proportion of adolescent homicide victimizations that involve firearms. At the same time, such policies may curb the rising presence of firearms in toddler and child homicide victimizations.

The findings should be weighed alongside known limitations of the SHR. First, the SHR uses a crude race variable that limits racial classifications to categories of Black, White, and victims of “other race,” and does not provide reliable measures of victim ethnicity. Second, the SHR contains limited information on the precise contexts in which homicides occurred (e.g., home, schools, public parks). Such information would further our understanding of pediatric killings and aid prevention efforts. Third, in 2021, the FBI moved its data collection program to the National Incident-Based Reporting System (NIBRS). While a version of the SHR data collection strategy was continued under NIBRS, low participation rates by law enforcement agencies has increased the amount of missing data. The multiple imputation procedure was therefore discontinued. Finally, the analysis is limited by missing data, particularly on perpetrator characteristics. As with past studies, our design attempts to account for missingness through a validated multiple imputation procedure [19].

It is important to note that much of the pediatric homicide literature focuses on the burden of firearm homicides [81–83]. However, with a strong focus on firearm homicides, the literature excludes considerable proportions of infant, toddler, and child victims who were not killed with firearms. The research portfolio aiming to address pediatric homicide as a preventable public health challenge must be inclusive of, but not exclusive to, firearm homicide outcomes.

## Conclusions

Numerous epidemiological studies offer critical insights into the characteristics of homicide victims. The current study builds on this work by offering descriptive information about the perpetrator characteristics and firearm use in pediatric homicide in the US over the past forty years. Findings reveal developmentally patterned trends in pediatric homicide circumstances, particularly with respect to perpetrators’ sex, age, relationship to victim, and firearm use. To prevent the future victimization of infants and toddlers, children, and adolescents, policy and program interventions must be developmentally sensitive, as well as considerate of the sex and race disparities embedded within.

## Data Availability

The Supplementary Homicide Reports (SHR) are publicly available under the Uniform Crime Reporting Program Data Series in the ICPSR repository, [https://www.icpsr.umich.edu/web/ICPSR/series/57]. The multiply imputed (SHR) version used in this work is available upon request from Prof. James Alan Fox at j.fox@northeastern.edu.

## Abbreviations

FBI: Federal Bureau of Investigation
NVDRS: National Violent Death Reporting System
NVSS: National Vital Statistics System
SHR: Supplementary Homicide Reports
UCR: Uniform Crime Reporting
US: United States
